# Intracranial ependymoma and secondary hydrocephalus: a rare case report in a Camel (*Camelus bactrianus*)

**DOI:** 10.3389/fvets.2026.1798157

**Published:** 2026-04-10

**Authors:** Luca Spadotto, Giovanni Martino, William Magnone, Camillo Sandri, Tommaso Banzato, Tommaso Gerussi, Massimo Castagnaro, Cinzia Centelleghe

**Affiliations:** 1 Department of Comparative Biomedicine and Food Science, University of Padua, Legnaro, Italy; 2 Immersive Parks, Natura Viva, Bussolengo, Italy; 3 Department of Animal Medicine, Production and Health, University of Padua, Legnaro, Italy

**Keywords:** brain, camel, ependymoma, neoplasia, neuropathology

## Abstract

Intracranial ependymomas are rare neuroepithelial tumors that arise from the ependymal lining of the ventricular system. Although sporadically reported in domestic species, primary brain tumors remain exceptionally rare in Old and New World camelids. This report describes the clinico-pathological characteristics of an intracranial ependymoma associated with secondary hydrocephalus in an adult female camel (*Camelus bactrianus*). The animal presented with progressive neurological signs, including a unilateral circling gait and ataxia. Computed tomography (CT) revealed asymmetric enlargement of the lateral ventricles with a mass effect. Post-mortem findings were characterized by a well-circumscribed, expansive intraventricular mass obstructing the left lateral ventricle and causing ventricular dilatation. Histologically, the neoplasm consisted of polygonal cells arranged in layers and perivascular pseudorosettes, with occasional true rosettes. It contained multifocal areas of necrosis, affecting approximately 25% of the mass, and was surrounded by multinucleated giant cells. Immunohistochemically, tumor cells showed strong and diffuse cytoplasmic positivity for vimentin, while glial fibrillary acidic protein (GFAP) expression was sparse and showed scattered cytoplasmic positivity in neoplastic cells. These findings confirmed the diagnosis of ependymoma. To the authors’ knowledge, this is the first reported case of intracranial ependymoma in a Bactrian camel.

## 1 Introduction

The ependyma consists of a monolayer of cuboidal, ciliated epithelial cells lining the inner surface of the central nervous system from the lateral ventricles to the filum terminale (1). These cells regulate the essential exchange at the interface between the brain interstitial fluid (BIF) and the cerebrospinal fluid (CSF) (2). In humans, the cIMPACT-NOW update 7 and the 2021 World Health Organization (WHO) classification of central nervous system (CNS) tumors classify ependymomas (EPNs)—neoplasms of ependymal cells—based on several factors, including anatomical location and histological, molecular, and epigenetic features (3, 4). EPNs are rare neuroepithelial tumors. In humans, these neoplasms account for 1.8% of all primary brain cancers and primarily affect children and young adults (5). Macroscopically, they are typically pinkish-gray and range from gelatinous to firm in consistency. Histologically, ependymomas consist of uniform, round-to-oval cells with speckled chromatin, arranged in characteristic perivascular pseudorosettes and true rosettes, with sharply demarcated margins and occasional necrosis, calcifications, or cystic changes (6). Tumor growth within the cerebral ventricles or spinal canal often results in the obstruction of cerebrospinal fluid circulation, leading to the formation of secondary obstructive hydrocephalus (7).

In veterinary medicine, there is no dedicated classification of CNS tumors; therefore, veterinary pathologists use a low-grade and high-grade (anaplastic) system, similar to the outdated human model. The majority of studies in recent years have attempted to bridge this gap (8–10), but there is still a lack of overall consensus that would allow for the creation of a system similar to that of the WHO. On gross examination, ependymomas are slow-growing, expansile, and variably demarcated masses that compress and replace the surrounding neuroparenchyma, typically appearing gray and fleshy, with occasional hemorrhage, cavitation, and a frequent association with secondary hydrocephalus (11). On histological evaluation in animals, the tumor is densely cellular, with small, dark, regular nuclei and undistinguished cytoplasmic boundaries. The cells can organize into pseudo-rosettes around blood vessels and also into true rosettes with basally arranged nuclei and surface cilia (11). Rare cases have been described in veterinary medicine, mostly involving domestic animals such as cats (12, 13), dogs (14–16), horses (17, 18), cattle (19–21), and other species, such as the white-tailed deer and rats (22, 23).

The current literature reports few cases of neoplasia in Old World camelids (such as *C. dromedarius*, Linnaeus 1758), whereas the majority of cases are documented in New World Camelids, such as llamas and alpacas (24). In the current literature, epithelial, mesenchymal, round cell, and a few other embryonal origin neoplasms have been reported; a notable lack of reports concerning CNS neoplasia is evident, with only two instances of primary brain tumors (a thalamic histiocytic sarcoma and a fibroblastic meningioma) reported in two Bactrian camels (25). Since that report, no new cases of primary brain tumors in Old World camelids have been documented.

## 2 Case description

### 2.1 Clinical presentation

A 13-year-old female Bactrian camel (*Camelus bactrianus*), kept under human care in a zoological garden, presented with acute-onset left circling movements, difficulty stopping completely, and disorientation. The camel did not appear anxious or distressed but exhibited impaired vision and balance and occasionally bumped into obstacles. During the first standing sedation, a physical examination was performed, revealing no abnormalities in the ears, eyes, or oral cavity, except for a slight deviation and a head tilt to the left. Hematology revealed leukocytosis (24.40 G/L; reference range 8.6–16.5 G/L), and serum biochemistry revealed high levels of GLDH (21.00 U/L; reference range <8 U/L), AST (127.90 U/L; reference range 69–97 U/L) and magnesium (1.40 mmol/L; reference range 0.75–0.95 mmol/L). Hematological and biochemical analyses were performed by a commercial veterinary diagnostic laboratory (LABOKLIN GmbH & Co. KG, Steubenstaße 4, 97,688 Bad Kissingen, Germany). The results were interpreted according to the species-specific reference intervals provided by the laboratory. Based on these results, antibiotic therapy was started with ceftiofur (10 mg/kg IM) every 72 h. Due to the lack of improvement, a second standing sedation was performed 15 days later; however, leukocytosis persisted, and AST levels were only mildly decreased. Clindamycin was added to therapy, but 2 months after the initial presentation, the camel continued to present neurological signs, such as depression, ataxia, and clockwise circular gait. In November 2024, the camel was humanely euthanized because of poor prognosis, medical management concerns related to its chronic weight loss and persistent circling, and continued disorientation.

The animal was subsequently transported to the laboratories of the Department of Comparative Biomedicine and Food Science (BCA) at the University of Padua (Italy) for a standardized *post-mortem* examination, performed as previously described (26).

### 2.2 Post-mortem examination

On *post-mortem* evaluation, the animal was in a good nutritional state. The lungs appeared diffusely dark red with a slightly increased consistency. Lobulation was distinctly visible, with the presence of multifocal fibrin strands on the pleurae. Upon sectioning the parenchyma, whitish, foamy material was observed exuding from the cut surface. Two well-encapsulated nodular formations containing greenish, purulent material were observed: one approximately 1 cm in size at the proximal third of the tongue and another approximately 4 cm in size in the right submandibular region. Additionally, multifocal, coalescent, encapsulated nodular structures of varying sizes (5 mm to 2 cm), attributable to abscesses, were identified in the mucosa of the first gastric compartment. The jejunum was segmentally dark red and flaccid, with dark red to black contents. On sectioning, the mucosa appeared dark red to blackish, and bloody material was present within the lumen.

**Figure 1:**
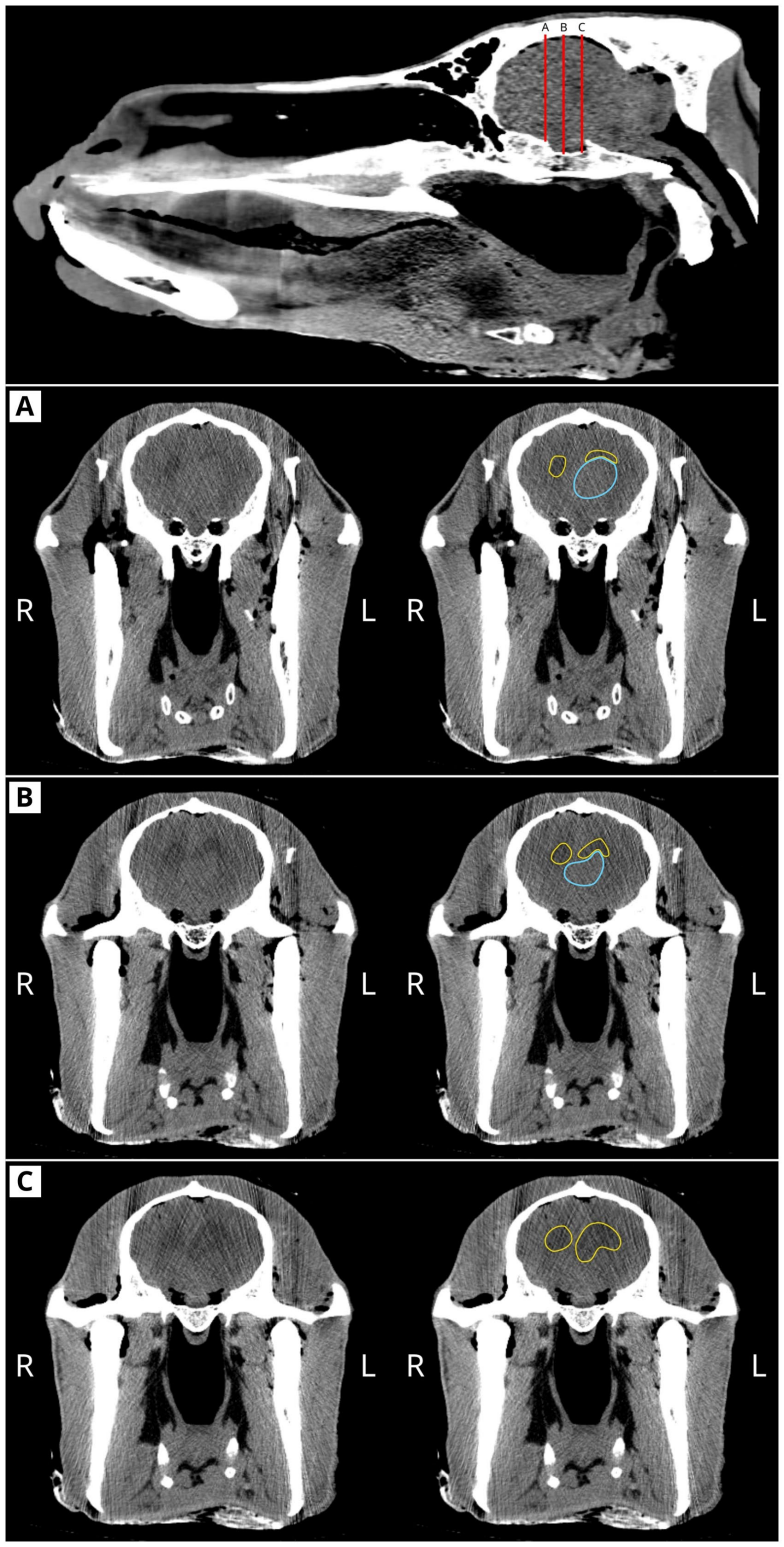
CT scan of the camel head. Top: the mid-sagittal plane of the head showing the coronal planes in rostro-caudal direction **(A–C)**. Coronal planes **(A–C)** present the left image without labels, while the right one presents the outline of the hypoattenuated areas (yellow outlines). From coronal plane **(A)** to coronal plane **(C)**, note the roundness of the right ventricle compared to the cranially flattened to enlarged caudal left ventricle. In coronal planes **(A,B)**, the light blue outlines delineate the intraventricular mass lesion.

During atlanto-occipital dislocation, an abundant amount of translucent cerebrospinal fluid (CSF), estimated at approximately 80 mL, was observed spilling through the *foramen magnum*. This finding, in association with the observed neurological symptoms, was central to the clinical decision to perform computed tomography (CT) imaging at the University Veterinary Teaching Hospital of the Department of Animal Medicine, Production, and Health (MAPS) at the University of Padua. After the removal of the head, CT imaging was performed using a Toshiba Asteion-S4 scanner with the following parameters: field of view (FOV) 380 mm, slice thickness 2 mm, tube voltage 120 kV, and tube current–time product 180 mAs. The CT examination revealed asymmetrically, moderately enlarged lateral ventricles, along with a well-defined intraventricular mass compatible with a neoplasm (Figure 1). A marked mass effect was observed on the left lateral ventricle, which appeared smaller than the contralateral ventricle.

**Figure 2:**
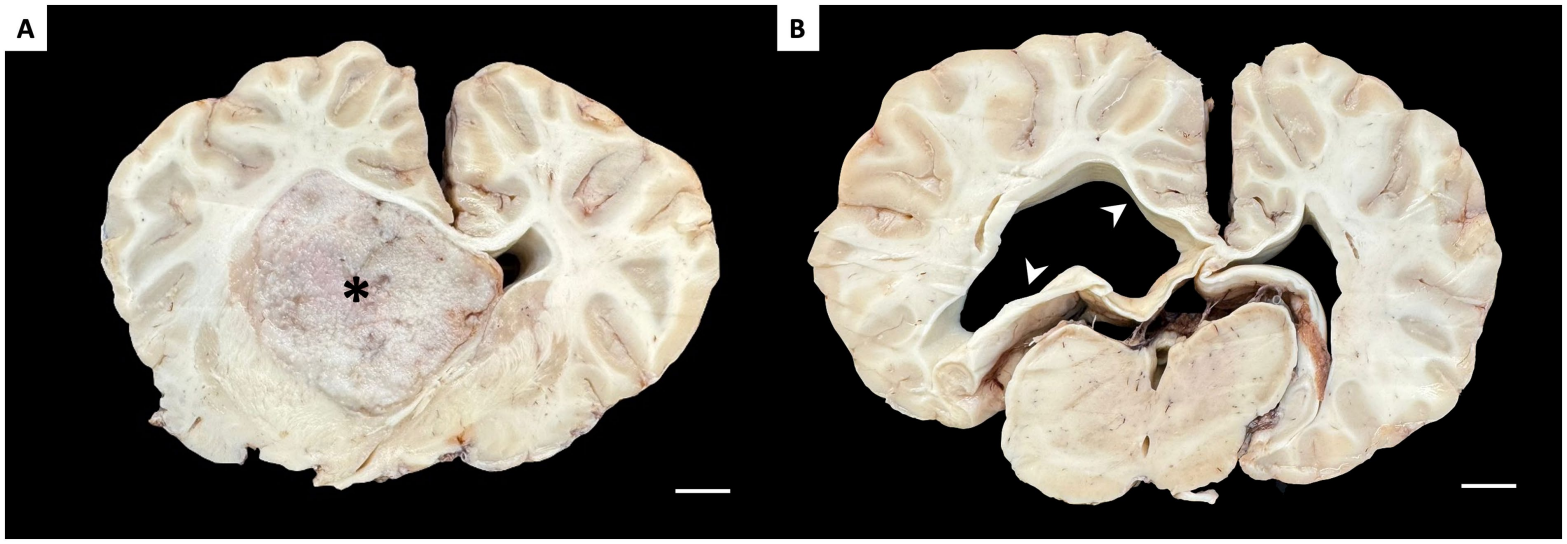
Macroscopic findings in coronal sections of formalin-fixed brain. **(A)** Section at the level of the basal ganglia. Filling the left lateral ventricle is a well-demarcated, tan mass (*), causing a midline shift. **(B)** Section at the level of the thalamus. The lateral ventricles are asymmetrically dilated, with more pronounced dilation in the ventricle ipsilateral to the tumor (arrowheads). Scale bar = 1 cm.

The brain was removed from the neurocranium and preserved in 10% neutral buffered formalin for 7 days. Then, the brain was inspected through a series of coronal cuts. Within the left ventricle, a well-defined, round mass measuring approximately 5 cm in diameter was identified (Figure 2A). The color and texture of the nodular tissue were notably distinct from the surrounding central nervous system, and the obstructive effect within the ventricular system was evident, resulting in a severe enlargement of the ipsilateral ventricle and a mild enlargement of the contralateral (Figure 2B), with subsequent hydrocephalus.

Other lesions included multifocal thyroid cysts of varying sizes (maximum 5 mm) and multifocal hepatic scars, likely of previous parasitological significance. These findings, while noteworthy, were considered incidental observations and unrelated to the primary pathological processes observed.

### 2.3 Histological, immunohistochemical, and ultrastructural findings

After necropsy, the main organs, including the liver, lungs, heart, gastric chambers, intestine, spleen, thyroid, various lymph nodes, kidneys, and CNS, were sampled for histopathological examination and preserved in 10% neutral buffered formalin.

The samples were then dehydrated and embedded in paraffin following standard procedures. Sections of formalin-fixed, paraffin-embedded (FFPE) tissues, measuring 3–5 μm thick, were obtained, mounted on glass slides, and stained with hematoxylin and eosin (H&E) using a routine protocol.

**Figure 3:**
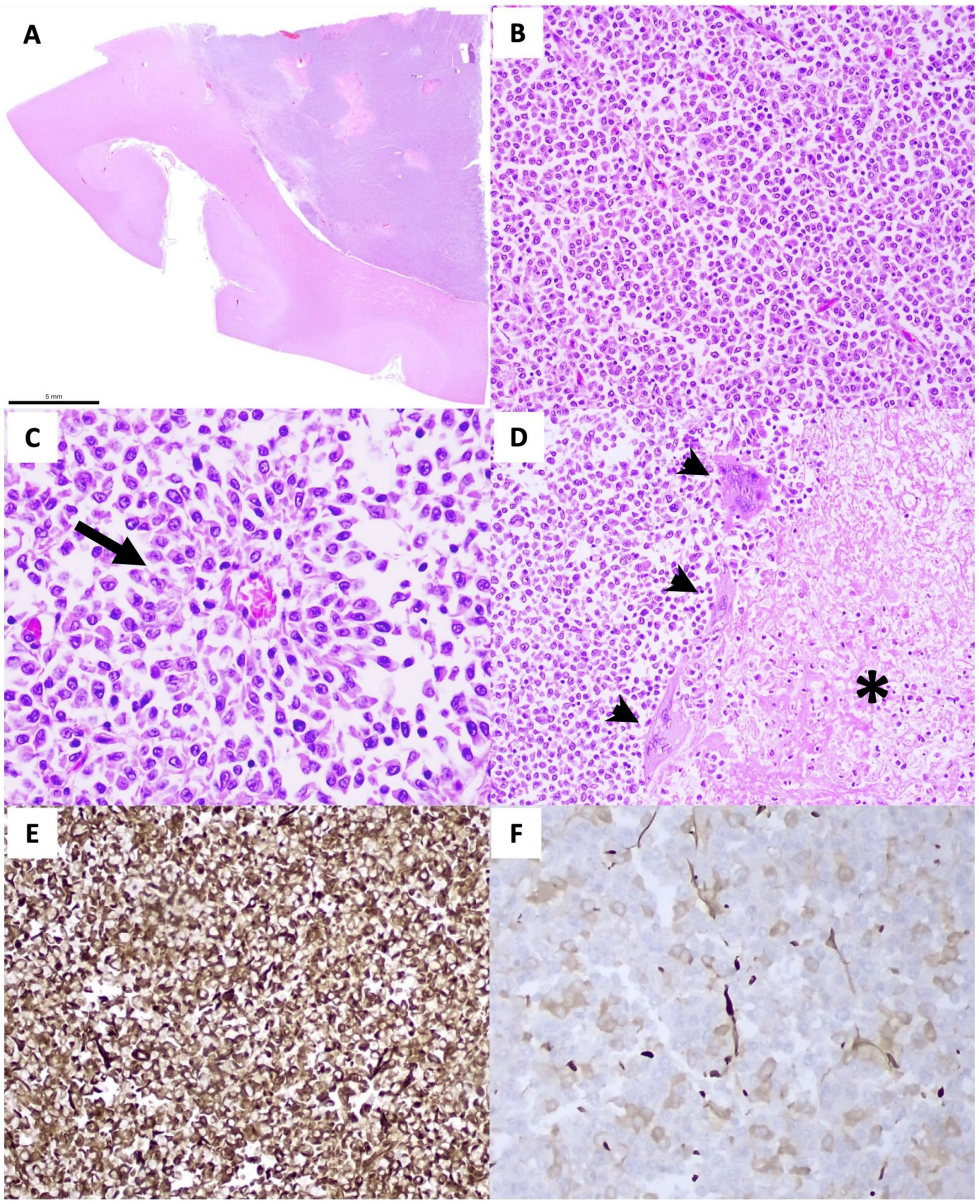
Histopathological **(A–D)** and immunohistochemical **(E,F)** evaluation. **(A)** A subgross view of the neoplastic mass expanding the lateral ventricle and focally infiltrating the neuroparenchyma. **(B)** The neoplastic cells are arranged in solid sheets in loose fibrovascular stroma (H&E, 20×) and occasionally in a palisade along the wall of a vessel, forming pseudorosettes (arrow) **(C)** (H&E, 40×). **(D)** Multifocally, there are areas of coagulative necrosis (asterisk) surrounded by multinucleated giant cells (arrowhead), (H&E, 20×). **(E)** Vimentin immunostaining demonstrates strong and diffuse cytoplasmic positivity in the majority of neoplastic cells, consistent with mesenchymal differentiation. **(F)** GFAP immunostaining shows scant and scattered cytoplasmic positivity in neoplastic cells.

On histologic examination, the left lateral ventricle was filled and expanded by a densely cellular, well-circumscribed, unencapsulated neoplasm (Figures 3A,B), composed of polygonal cells arranged in sheets, pseudorosettes, and occasionally true rosettes on a fine fibrovascular stroma (Figure 3C). Neoplastic cells had variably distinct cell borders and a small-to-moderate amount of eosinophilic, granular cytoplasm. Nuclei were round to oval, with finely stippled to coarsely granular chromatin and a variably distinct nucleolus. Anisocytosis and anisokaryosis were mild, and the mitotic count was 7 per 2.37 mm^2^. Scattered throughout the neoplastic cells were multifocal areas of coagulative necrosis, often surrounded by a variable number of multinucleated giant cells (Figure 3D). The latter were also rarely observed in other areas of the neoplasm. Neoplastic cells multifocally infiltrated the periventricular neuroparenchyma, and parenchymal blood vessels were multifocally cuffed by mononuclear inflammatory cells.

Immunohistochemical (IHC) analysis was performed using a semiautomatic immunostainer (Benchmark GX, Ventana, Tucson, AZ, USA) with an anti-Vimentin antibody (Clone V9, DAKO, Glostrup, Denmark; #M0725), which showed intense and diffuse cytoplasmic immunoreactivity in the neoplastic cells (Figure 3E). A canine mesenchymal tumor was used as an external positive control, and vascular endothelial cells in adjacent non-neoplastic camel brain tissue served as an internal positive control. Immunoreactivity with anti-glial fibrillary acidic protein (GFAP) antibody (Clone 6F2, Diagnostic BioSystems, Pleasanton, CA, USA; #MOB199-05) was scant and multifocal (Figure 3F). Normal canine brain tissue was used as an external positive control, and astrocytes in adjacent non-neoplastic camel brain tissue served as an internal positive control. For both IHC analyses, negative controls were performed by omitting the primary antibody.

**Figure 4:**
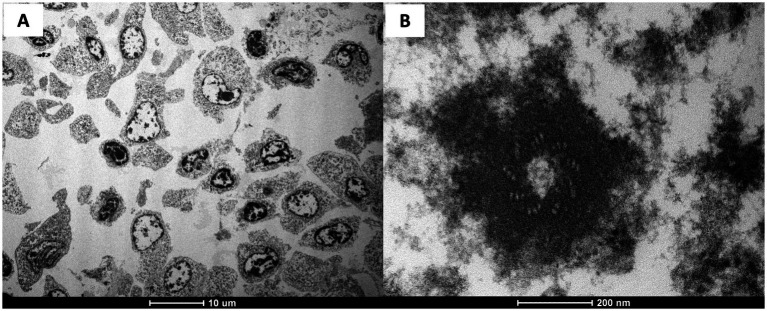
Ultrastructural examination **(A,B)**. **(A)** The TEM of the neoplastic cells reveals marked pleomorphism, irregular nuclear contouring, and prominent anisokaryosis; the cytoplasm is variably expanded with loss of structural orientation, and the cell membranes appear variably disrupted and poorly delineated. **(B)** A residual basal body can be identified by its radial arrangement of triplet microtubules, despite the surrounding markedly degraded cytoplasmic matrix.

Transmission electron microscopy (TEM) was performed on a formalin-fixed sample of the intracranial mass. A representative tissue fragment was post-fixed in osmium tetroxide, dehydrated, embedded in epoxy resin, and sectioned following routine protocols for archival materials. An ultrastructural examination revealed marked cellular atypia, including pleomorphism, anisocytosis, and anisokaryosis (Figure 4A), with scattered neoplastic cells displaying identifiable basal bodies (Figure 4B), although ciliary structures were largely absent.

## 3 Discussion

To our knowledge, this is the first reported case of ependymoma in a Bactrian camel (*C. bactrianus*, Linnaeus 1758). In the present case, the diagnosis of ependymoma was made based on pathological findings and the IHC profile of the proliferating cells. Other cases of ependymoma in veterinary species presented with clinical signs, including ataxia, depression, dysphagia, apathy, emaciation, drowsiness, gait abnormalities, such as monolateral and circling gait, fecal and urinary incontinence, incoordination, seizures, and various ocular signs, such as blindness, anisocoria, and mydriasis (27–32). The signs described in this case, monolateral circling gait, apathy, and ataxia, are compatible with mild nervous symptoms described in cases of intracranial ependymoma. Behavioral changes and neurological signs often result from inflammatory and degenerative processes secondary to neoplastic compression and invasion of brain tissue (33). It is well reported in the literature that structures causing compression within the cerebral ventricular system alter CSF flow, increasing ventricular volume due to cerebrospinal fluid accumulation (34), which in turn causes brain tissue compression, neuronal dysfunction, and ultimately neurological clinical signs (35). Intracranial ependymomas in animals are described as soft, round to oval pale masses within the ventricles (36) that can variably infiltrate and compress the adjacent parenchyma (14). In our case, the firm, oval mass filled the lateral ventricle and extended into the adjacent neuroparenchyma. Furthermore, an abundant volume of CSF accumulated in the ventricular system, suggesting underlying hydrocephalus.

Histomorphologically, ependymomas must be differentiated from choroid plexus tumors and primitive neuroectodermal tumors (31). Choroid plexus tumors, which frequently originate in the fourth ventricle, were excluded due to the absence of histological features such as vascular papillae and cuboidal or columnar epithelium (11, 31, 37). Neuroectodermal tumors are predominantly cerebellar in young animals, which is inconsistent with the animal’s age and tumor site in this case (38). The location and histologic features, including true and pseudorosettes, were most consistent with a diagnosis of ependymoma (11).

A definitive diagnosis of ependymoma requires IHC evaluation. Antibodies used to confirm the diagnosis of ependymoma are usually vimentin and GFAP (39). Consistent with previous reports of ependymoma in animals, the IHC results in this study showed that the vimentin labeling was strongly positive, whereas GFAP labeling demonstrated partial, inconsistent positivity. The partial positivity for GFAP could be attributed to several factors, such as the lack of antibody validation for the species studied or, as suggested by some studies in dogs (14, 16), a low degree of tumor differentiation. Moreover, the ultrastructural features demonstrated pronounced cellular atypia, including pleomorphism, anisocytosis, and anisokaryosis, which limited the recognition of morphological features typically associated with ependymal differentiation. Classical ependymal characteristics, such as well-organized apical specializations, microvilli, or cilia (40), were not evident. This lack of identifiable structures may reflect two non-mutually exclusive factors. On one hand, primary fixation in neutral buffered formalin is known to compromise the preservation of membranous and surface-associated elements, often resulting in partial or complete loss of ciliary profiles during processing (41). On the other hand, the high degree of anaplasia observed histologically is consistent with a poorly differentiated neoplasm, in which ultrastructural markers of lineage are frequently diminished or absent (42). The presence of occasional basal bodies suggests that a residual ciliogenic apparatus was present but either insufficiently preserved or intrinsically reduced.

Importantly, despite the limited ultrastructural evidence, the diagnosis of ependymoma is strongly supported by the anatomical location of the mass, the histological identification of rosettes and pseudorosettes, and the immunohistochemical profile, including partial GFAP positivity. In addition, although the TEM results were not optimal, the ultrastructural features that could be assessed remain consistent with this diagnosis and reinforce the overall interpretation.

## 4 Conclusion

The gross, histological, and immunohistological features of this ventricular mass in a Bactrian camel are consistent with the diagnosis of ependymoma. This neoplasm has not been previously reported in Old World camelids.

## Data Availability

The original contributions presented in the study are included in the article/supplementary material, further inquiries can be directed to the corresponding author.
